# CBCT-Based Dose Monitoring and Adaptive Planning Triggers in Head and Neck PBS Proton Therapy

**DOI:** 10.3390/cancers15153881

**Published:** 2023-07-30

**Authors:** Keaton Reiners, Roi Dagan, Adam Holtzman, Curtis Bryant, Sebastian Andersson, Rasmus Nilsson, Liu Hong, Perry Johnson, Yawei Zhang

**Affiliations:** 1 University of Florida Health Proton Therapy Institute, Jacksonville, FL 32206, USA; keatonreiners@ufl.edu (K.R.); rdagan@floridaproton.org (R.D.); cbryant@floridaproton.org (C.B.); pbjohnson@floridaproton.org (P.J.); 2 Medical Physics Graduate Program, University of Florida College of Medicine, Gainesville, FL 32610, USA; 3 Department of Radiation Oncology, University of Florida College of Medicine, Gainesville, FL 32610, USA; 4 Department of Radiation Oncology, Mayo Clinic, Jacksonville, FL 32224, USA; holtzman.adam@mayo.edu; 5 RaySearch Laboratories, SE-103 65 Stockholm, Sweden; sebastian.andersson@raysearchlabs.com (S.A.); rasmus.nilsson@raysearchlabs.com (R.N.); 6 Ion Beam Applications S.A., 1348 Louvain-la-Neuve, Belgium; liu.hong@iba-group.com

**Keywords:** proton adaptive therapy, PBS, cone-beam CT, head and neck, synthetic CT

## Abstract

**Simple Summary:**

Head and neck cancer patients require adaptive radiation therapy due to aggressive tumor response, changes in anatomy, and difficulties with setup reproducibility. Additionally, protons are more sensitive to patient setup uncertainty and anatomical change than photons. At our institution, verification CTs are often utilized to evaluate changes in anatomy and dose coverage during the treatment course. Nevertheless, they are limited by low frequency and an inability to detect changes in treatment setup. Furthermore, our current clinical workflow does not have definitive thresholds for adaptive therapy plan review, meaning every verification CT must be reviewed by a physician and physicist. We analyzed the feasibility of higher-frequency dose monitoring and triggering a plan review for adaptive proton therapy by recalculating the plan dose on synthetic CTs produced from daily patient setup cone-beam CTs. Plan review triggers were established following the analysis of cohort data. Two synthetic CT algorithms were developed and evaluated. Both resulted in images that outperformed the verification CTs in dosimetric accuracy. A more efficient and accurate adaptive proton therapy workflow was successfully established.

**Abstract:**

Purpose: To investigate the feasibility of using cone-beam computed tomography (CBCT)-derived synthetic CTs to monitor the daily dose and trigger a plan review for adaptive proton therapy (APT) in head and neck cancer (HNC) patients. Methods: For 84 HNC patients treated with proton pencil-beam scanning (PBS), same-day CBCT and verification CT (vfCT) pairs were retrospectively collected. The ground truth CT (gtCT) was created by deforming the vfCT to the same-day CBCT, and it was then used as a dosimetric baseline and for establishing plan review trigger recommendations. Two different synthetic CT algorithms were tested; the corrected CBCT (corrCBCT) was created using an iterative image correction method and the virtual CT (virtCT) was created by deforming the planning CT to the CBCT, followed by a low-density masking process. Clinical treatment plans were recalculated on the image sets for evaluation. Results: Plan review trigger criteria for adaptive therapy were established after closely reviewing the cohort data. Compared to the vfCT, the corrCBCT and virtCT reliably produced dosimetric data more similar to the gtCT. The average discrepancy in D99 for high-risk clinical target volumes (CTV) was 1.1%, 0.7%, and 0.4% and for standard-risk CTVs was 1.8%, 0.5%, and 0.5% for the vfCT, corrCBCT, and virtCT, respectively. Conclusion: Streamlined APT has been achieved with the proposed plan review criteria and CBCT-based synthetic CT workflow.

## 1. Introduction

Head and neck cancer patients are strong candidates for adaptive therapy due to complex target volumes, multiple critical structures close to treated areas, and aggressive tumor response that results in significant changes to target and organ-at-risk (OAR) anatomy [1]. Previous studies have shown that adaptive radiation therapy can improve target coverage, OAR sparing, plan quality, and quality of life for head and neck cancer patients [2,3,4,5,6]. Adaptive radiation therapy has also demonstrated a significant correlation with the disease-free survival and overall survival of head and neck cancer patients [1,4]. Proton therapy offers certain dosimetric advantages over photon therapies for head and neck cancer patients due to the intrinsic beam characteristics of proton therapy, such as minimal lateral penumbra and rapid distal fall-off. Compared to advanced photon treatments, the use of proton therapy has been shown to decrease dose to normal tissue, improve sparing to nearby organs, reduce integral dose, and decrease overall toxicities in head and neck cancer treatment [7,8,9,10,11,12,13].

Though the benefits of proton therapy are promising, it still presents multiple challenges in the adaptive setting. While more advanced planning techniques, such as robust optimization or careful consideration of the beam path, may alleviate some concerns regarding range and dose uncertainty, protons remain more sensitive to patient setup and anatomy changes than photons. The steep dose gradients allow for highly conformal dose distributions but can result in more severe underdosing of targets and/or overdosing of OARs if patient anatomy and/or setup geometry deviate from the initial plan. Stützer et al. [14] reported that recalculated intensity-modulated proton therapy (IMPT) doses showed more significant degradation throughout treatment than photon intensity-modulated radiation therapy (IMRT) plans. Furthermore, reliable proton dose calculation requires accurate stopping power ratios (SPR) obtained from computed tomography (CT) numbers; therefore, calculating a proton dose distribution is more sensitive to CT number inaccuracy and imaging artifacts [15].

**Figure 1:**
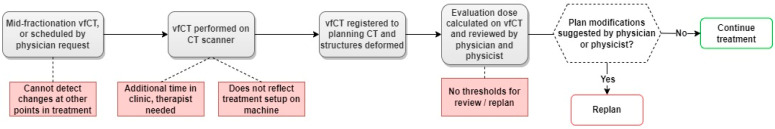
Clinical workflow currently used at our institution for evaluating the need for adaptive proton therapy throughout the course of treatment (red boxes indicate limitations in the current workflow).

In our current clinical practice, weekly or mid-fractionation verification CTs (vfCT) are often acquired for head and neck cancer patients to evaluate the need for adaptive proton therapy. The vfCT is rigidly registered to the planning CT (pCT), and the dose is recalculated on the vfCT with initial planning contours deformed to the new image set. The workflow currently used at our institution is outlined in Figure 1 and has multiple notable limitations. One such limitation is an inability to detect the need for adaptive therapy at times in the treatment course that do not correspond with a verification CT. This may lead to overdosing of surrounding OARs, or underdosing of target volumes, due to the aggressive tumor response seen in head and neck cancer patients. A previous study demonstrated significant changes in target volume for head and neck cancer patients within the first ten days of treatment, with a mean volume reduction of 16.4% for the gross target volume (GTV) and 15.4% for the primary clinical target volume (CTV) [16]. These changes in volume have been associated with changes in dosimetry and plan quality, resulting in lower doses and a reduction in homogeneity to the target volumes [17,18,19]. Also, the current vfCT method is unable to detect setup issues in daily treatments due to the verification CT being acquired on a CT simulator without the ability to correct setup discrepancies from the planned position. Additionally, our current clinical practice does not have defined dosimetric thresholds for triggering a plan review to evaluate the need for adaptive therapy, which means that physicians and physicists must review every vfCT to determine if adaptation is necessary. Moreover, the current workflow requires additional patient appointments, clinical resources, and staff.

Pre-treatment cone-beam computed tomography (CBCT) images are often acquired to set the head and neck cancer patient up for proton therapy. Due to the inaccuracy of the CT numbers, the use of CBCT has been limited to assisting with daily patient setup. Various methods of utilizing CBCTs for plan recalculation have been explored, such as deforming the planning CT to the CBCT using deformable image registration [20,21,22,23,24,25,26,27], scatter correction methods [26,28,29], image correction utilizing deep convolution neural networks (DCNN) [27,30,31,32], histogram matching [33], and corrections via look-up tables [25]. In this study, we propose a CBCT-based dose monitoring and adaptive planning trigger process for head and neck adaptive proton therapy, allowing for more frequent dose verifications and the ability to recognize changes in both anatomy and setup geometry.

There are three principal aims of this work: first, defining target coverage thresholds for triggering further plan review for adaptive proton therapy that can be implemented into our institution’s workflow by analyzing dosimetry changes for head and neck cancer patients treated with pencil-beam scanning (PBS) proton therapy; second, evaluating the dose calculation accuracy of two synthetic CT algorithms integrated into a commercial treatment planning system (TPS); third and final, developing and implementing an automated, highly reliable workflow to replace the current verification CT workflow used clinically for head and neck cancer proton treatment.

## 2. Materials and Methods

### 2.1. Patient Selection and Image Data

Eighty-four consecutive patients with head and neck cancer were retrospectively enrolled in this study under a protocol (UFPTI2210-HNX08) approved by the institutional review board of the University of Florida. All patients were previously treated on a Proteus^®^ONE PBS gantry (Ion Beam Applications SA, Louvain-la-Neuve, Belgium) between January 2020 and January 2022. Patient simulation scans were performed on a Philips Big Bore CT (Philips Healthcare, Amsterdam, the Netherlands). All patients in this study had a vfCT performed on the same scanner as the planning CT during the treatment course. CBCT images were acquired for daily setup with the gantry-mounted CBCT image system on Proteus^®^ONE. Further details of the images obtained are outlined in Table 1.

### 2.2. Treatment Planning

The originally treated PBS plans were optimized and calculated in the RayStation version 9B (RaySearch Laboratories AB, Stockholm, Sweden) treatment planning system. All patients were treated using a simultaneous integrated boost (SIB) prescription, resulting in high-risk and standard-risk target volumes. High-risk volumes received 2 Gy/fraction, while standard-risk volumes received 1.6–1.8 Gy/fraction for 30–35 fractions. All 84 patients had a high-risk CTV and standard-risk CTV defined. Typical beam arrangement for head and neck planning at our institution includes one anterior beam and two posterior oblique beams, or one posterior beam and two anterior oblique beams. Targets were robustly optimized with an isotropic shift uncertainty of 3 mm applied to each beam individually with the systematic density uncertainty set to 3.5%. Dose calculation was performed using the Monte Carlo algorithm with a maximum allowed statistical uncertainty of 0.5% and a dose calculation grid resolution of 2 × 2 × 2 mm^3^. All patients were transferred from the planning version of RayStation to a research version of RayStation 12A where verification doses were recalculated on generated images. While performing a robust evaluation on the generated images was not within the scope of this study, all other calculation parameters were kept identical to the original treatment plan calculation.

### 2.3. Image Set Overview

Each patient begins with three image sets. The pCT contains the original anatomy and contours on which the delivered plan was designed. A vfCT is a diagnostic-quality CT image taken during treatment, typically near the middle of the course of therapy. The last image set is the CBCT, specifically the CBCT image obtained on the same day as the verification CT. These three image sets allow for the generation of three additional images by utilizing proprietary algorithms within RayStation [34]: the “ground truth CT” (gtCT), “corrected CBCT” (corrCBCT), and the “virtual CT” (virtCT). The methods for producing the three additional images were previously outlined and investigated by Thing et al. [35] for use in photon therapy, and are further described in the following sections. The methods are intended to be approved for proton therapy and released for clinical use in an upcoming version of RayStation.

### 2.4. Ground Truth CT (gtCT) and Defining Adaptive Therapy Triggers

The gtCT is generated by deforming the vfCT to the same-day CBCT. The high-quality vfCT image taken on the same day of treatment is highly representative of the anatomy treated for that particular fraction and will possess minimal artifacts. Deforming the image to the same-day CBCT removes setup differences between the vfCT positioning in the scanner and the actual treatment position. This results in the most accurate possible dose calculation for the given fraction and will act as the reference dose when comparing the accuracy of other image sets. The recalculated plan dose on the gtCT was also used for evaluating the dosimetric changes and loss of target coverage at the time of the vfCT for the patient cohort. Compared to a workflow that requires scheduled vfCT scans, the CBCT-based synthetic CT workflow will allow for more frequent dose monitoring, such as daily dose monitoring. In a workflow with high-frequency dose monitoring, it is impractical for every dose evaluation to be reviewed by a physician and physicist, and dosimetric thresholds must be established for triggering further plan review. A team of physicians specializing in head and neck cancer was consulted to review the cohort data to establish clinical recommendations and thresholds for triggering further plan review for adaptive proton therapy.

### 2.5. Corrected CBCT (corrCBCT)

**Figure 2:**
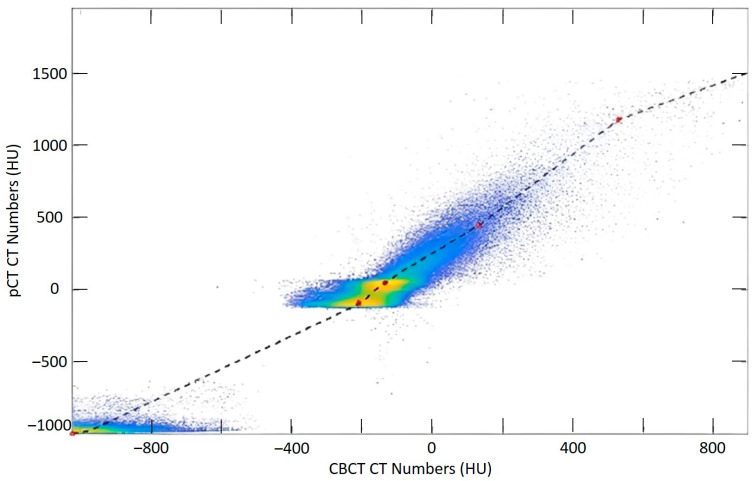
A joint histogram and the piecewise linear conversion function between the pCT and CBCT were generated for mapping gray value intensities in the CBCT to corresponding CT numbers in the pCT. Red points indicate junctions in the piecewise function.

**Figure 3:**
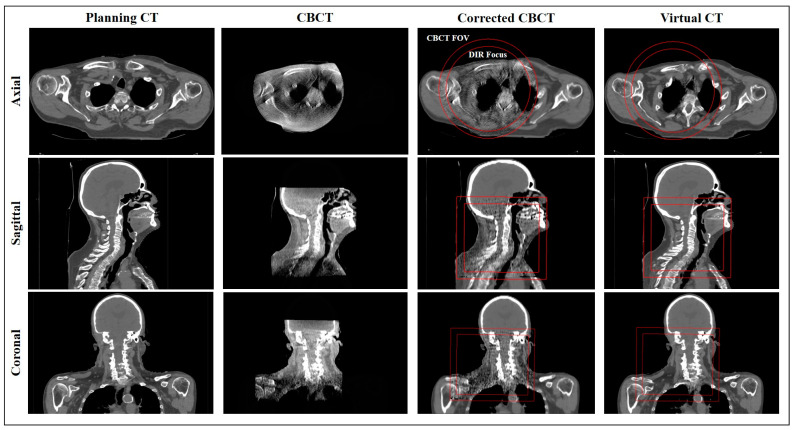
A patient example showing axial, sagittal, and coronal views for the pCT, CBCT, corrCBCT, and virtCT. Two ROIs are demonstrated on the axial view of the corrected CBCT: the field of view ROI and the field of view ROI retracted 2 cm, which is used to focus the deformable image registration.

The corrCBCT is generated using multiple steps in an iterative image correction process. The pCT is aligned to the CBCT using deformable image registration (DIR) and, thereafter, a joint histogram of the image intensities is generated. Then, points on the histogram corresponding to different tissue classes are identified. A piecewise linear conversion function is produced to convert gray values in the CBCT to corresponding CT numbers in the pCT. An example of the joint histogram and conversion function is shown in Figure 2. After applying the conversion function, the images can be quantitatively analyzed for artifact correction, using a method based on the work of Marchant et al. [36]. An artifact correction map is generated to identify and remove low-frequency artifacts in the CBCT, utilizing a difference map between the pCT and CBCT combined with a low-pass filter. The intensity conversion and artifact correction steps are performed iteratively until a final solution is converged upon. Outside of the CBCT field of view, the anatomy in the corrCBCT is extended by deforming and mapping over the CT numbers from the pCT. This transition from the corrected CBCT image to pCT outside the field-of-view (FOV) can be seen in Figure 3.

### 2.6. Virtual CT (virtCT)

The virtCT is generated by deforming the pCT to the CBCT, then correcting for low-density anatomical differences found when comparing the image to the corrCBCT. The virtCT utilizes the CT numbers from the planning CT, but if a density change (Δρ > 0.3 g/cm^3^) is noted in a particular region between the virtCT and the corrCBCT, the CT number from the corrCBCT replaces the value from the pCT. This allows the virtCT to accurately represent changes in low-density anatomies, such as nasal filling, fluid buildup, or air cavities. This masking is only performed in regions where one of the images has a density <0.6 g/cm^3^, in order to avoid modifying or duplicating high-density structures. An example of the virtCT is shown in Figure 3.

### 2.7. Deformable Image Registration

The DIR was performed with the Anatomically Constrained Deformation Algorithm, known as the “ANACONDA” method. The ANACONDA method is a hybrid solution for DIR that combines the benefits of geometric and intensity-based methods for deforming images [37]. The FOV for CBCT images is smaller than that of the pCT, so the DIR was limited to a focused region. This region was determined by creating a region of interest (ROI) in the treatment planning system, defined as the entire CBCT field of view, then retracted 2 cm to avoid edge effects in the DIR. The ROI used for focusing the DIR can be seen in Figure 3. The deformations, registrations, and deformable contour mapping steps were all performed with a script to ensure minimal human influence on the results of the generated images and evaluation doses. Registrations and contours were checked to ensure they were reasonable, but no manual editing was performed.

### 2.8. Dose Metrics and Statistical Testing

Target coverage was computed and evaluated on the initial plan, gtCT, vfCT, corrCBCT, and virtCT. The primary target coverage metric utilized was D99, with the relative dose covering 99% of the target volume for the high-risk and standard-risk CTV. Dose–volume histograms (DVH) of the plan dose and evaluation doses were computed in the treatment planning system and then compared using an in-house script. To evaluate the dosimetric accuracy of the corrCBCT and virtCT compared to the vfCT, which is currently used clinically, the evaluation target coverage dose metrics were compared against the gtCT. Statistical significance was tested using the Wilcoxon signed-rank test, and the results were considered significant for *p* < 0.05.

### 2.9. CBCT Reconstruction Parameter Testing

Voxel and grid dimensions for the CBCT images used in this study are outlined in Table 1. To examine possible CBCT reconstruction effects on the synthetic CT algorithms, selected patients were evaluated with CBCT images reconstructed at various voxel sizes, slice thicknesses, and grid dimensions. The different CBCT reconstructions did not reveal any significant impact on the resulting evaluation doses.

## 3. Results

### 3.1. Target Coverage Loss and Adaptive Planning Trigger Criteria

**Figure 4:**
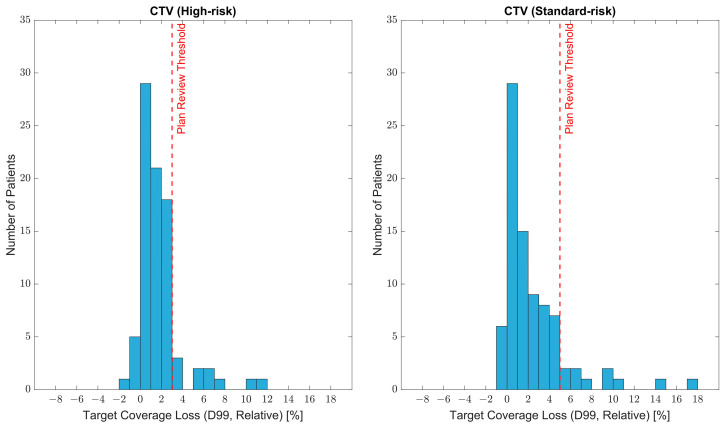
Distribution of target coverage loss (D99, relative), based on gtCT evaluation doses, for high-risk CTV and standard-risk CTV for the 84 patients analyzed. Positive values correspond to a loss of coverage. The figures also indicate the recommended threshold for adaptive therapy plan review.

The distributions of coverage loss for the target volumes analyzed are shown in Figure 4. At the time of the verification CT scan, based on the gtCT evaluation dose, the average decrease in D99 for the high-risk CTV was 1.8%, while the average decrease in D99 for the standard-risk CTV was 2.4%. Cohort data and patient plans were carefully reviewed with a team of physicians at our institution, and a consensus was formed for target coverage thresholds that would trigger further plan review in our adaptive therapy workflow. A patient would be considered for adaptive therapy for a D99 decrease of >3% on high-risk CTVs or >5% on standard-risk CTVs. Based on evaluation doses computed on the gtCT, 10/84 patients would trigger a plan review due to coverage loss on the high-risk CTV and 10/84 patients would trigger a plan review due to coverage loss on the standard-risk CTV. Multiple patients, however, had coverage loss beyond the threshold for both the high-risk and standard-risk CTV, resulting in a total of 16/84 (19%) of patients triggering a plan review based on the established criteria.

### 3.2. Comparing Dose Accuracy of Synthetic CTs and Verification CT

**Figure 5:**
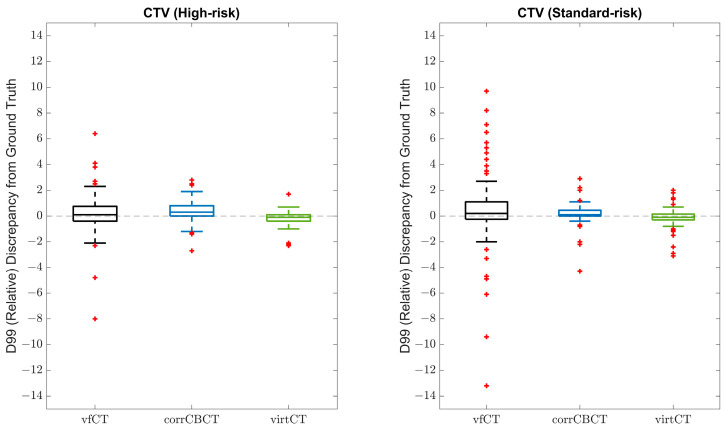
Comparison of vfCT, corrCBCT, and virtCT accuracy for target coverage relative to the gtCT. This is plotted for high-risk CTV and standard-risk CTV. The coverage metric analyzed was relative dose at 99% volume (D99). Unless otherwise specified, all box plots have the same format: the center line is average, box edges are interquartile, whiskers are data within the standard deviation, and points beyond whiskers are outliers. The closer to zero, the better agreement between the calculated dose and the ground truth dose.

The comparison of calculated target coverage accuracy between the corrCBCT, virtCT, and vfCT compared to the gtCT is shown in Figure 5 and summarized in Table 2. The coverage metrics computed on the corrCBCT and virtCT were significantly more similar to the dose on the gtCT relative to the metrics calculated on the vfCT for both high-risk CTVs and standard-risk CTVs.

### 3.3. Established Workflow for Dose Monitoring on CBCT-Based Synthetic CTs

**Figure 6:**
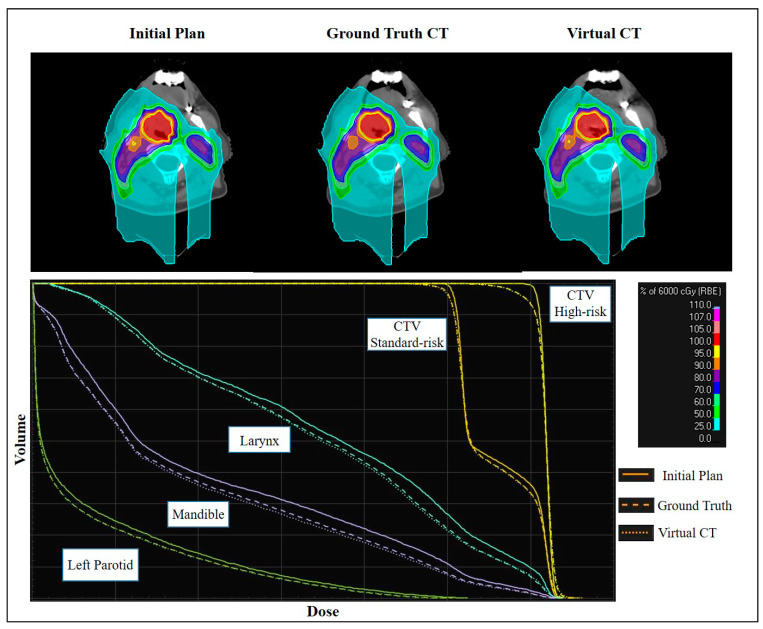
Demonstration of the dose comparisons within the treatment planning system. A comparison of the initial plan dose and evaluation doses computed on the gtCT and virtCT are shown. The virtCT closely agrees with the gtCT in terms of reflecting dosimetry changes to the targets and OARs.

**Figure 7:**
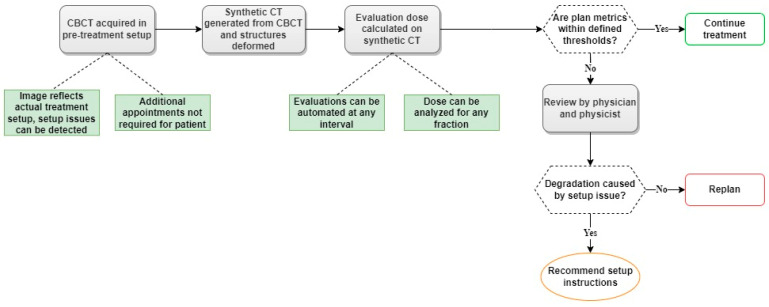
Proposed CBCT-based synthetic CT workflow for evaluating the need for adaptive proton therapy throughout the course of treatment (green boxes indicate potential advantages of proposed workflow).

A workflow was successfully established for generating both synthetic CTs, the corrCBCT and virtCT, for dose monitoring and adaptive therapy evaluation using scripting within the treatment planning system. Once the desired CBCT image is imported into the treatment planning system, the script can automatically generate the corrCBCT and virtCT, map deformed structures onto each image set, and recalculate the initial plan dose on each image. The established workflow allows dose visualization and DVH comparisons between images within the treatment planning system. An example of this is shown in Figure 6. Additionally, a complete clinical workflow has been proposed as a replacement for the current workflow previously discussed and outlined in Figure 1. The new workflow is shown in Figure 7.

## 4. Discussion

### 4.1. Cohort Findings

Following a review of the dosimetric data from the cohort, recommended thresholds for triggering an adaptive therapy plan review were established based on target coverage. Experience at our institution has shown that a loss of target coverage is the primary reason for triggering adaptive therapy, and the experiences of other institutions support this. A review article by Huiskes et al. [2] found that for all studies reviewed for adaptive proton therapy in head and neck cancer patients, adaptive therapy was indicated due to target coverage deterioration and was never indicated due to doses to OARs. This was not true for adaptive therapy with photons, and this difference is likely due to the improved initial OAR sparing seen in proton therapy [2]. Based on the established thresholds for plan review (D99 decrease of >3% for high-risk CTVs and >5% for standard-risk CTVs), 19% of patients included in the study would trigger a physician and physicist review for adaptive proton therapy in our new workflow. This plan review rate does not directly translate into a replanning rate, as our proposed workflow allows for setup errors to be detected and, therefore, setup corrections to be implemented that lead to improved dosimetry without the need for replanning. Additionally, the plan review rate cannot be regarded as predictive of a replanning rate. In our cohort analysis, only one dose evaluation, acquired near the middle of the treatment course, was considered for each patient. The new workflow will allow for more frequent evaluations, which may lead to a plan review and possible plan adaptation at various points in the treatment course that would not have been detected by the lower-frequency evaluations in the prior workflow. Thus, our cohort analysis was simply used for clinicians to establish clinical context for target coverage loss that is typically experienced and to develop thresholds for plan review. Rates for replanning and adaptive therapy resulting from the new workflow will be investigated further following clinical implementation. Replanning rates vary widely in single-institution studies and are likely due to different criteria for replanning. Replanning rates for head and neck cancer patients treated with IMRT range from 21 to 65%, while replanning rates for patients treated with IMPT range from 8 to 63% [19,21,38,39,40].

**Figure 8:**
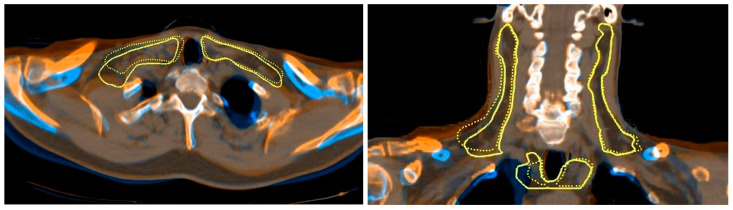
Axial (**left**) and coronal (**right**) views of the fusion for a patient’s planning and ground truth CTs. The solid-line contour represents the original planning contour and the dotted line represents the deformed contour to the treatment position on the ground truth CT.

The cohort analysis revealed more significant target coverage loss on standard-risk CTVs than on high-risk CTVs, on average. The distribution of coverage loss was also wider for standard-risk CTVs, with a |ΔD99| interquartile range of 0.5–3.2%, compared to 0.7–2.4% for high-risk CTVs. After a close review of patient registrations, it was concluded that the increased dose deterioration on the standard-risk CTVs was due to setup variations that impact this volume more than the high-risk volume. The standard-risk volumes in head and neck cancer patients typically follow nodal chains into the neck’s inferior portion and the superior part of the thorax. A study by Chen et al. [41] demonstrated that setup errors in the lower neck have more significant variance and result in larger geometric shifts than setup errors in the upper neck and head. Small changes in shoulder positioning during setup can result in substantial dose differences in the treated volumes. An example of a modified shoulder position during treatment is shown in Figure 8, and was seen in multiple patients to varying degrees. The targets with the greatest coverage loss were standard-risk CTVs that shifted due to a change in shoulder position.

### 4.2. Image Accuracy

Multiple studies have explored the synthetic CT methods used in this paper. Thing et al. [35] investigated the methods used for photon 3D-conformal and VMAT plan recalculation for breast, prostate, anal/rectal, and lung cancer patients. When comparing the gamma pass rates for the corrCBCT and virtCT, Thing et al. found the virtCT to have significantly (*p* < 0.05) higher pass rates than the corrCBCT for breast, prostate, and anal/rectal sites but did not show the significance for lungs. Hamming et al. [42] investigated the methods for plan recalculation for breast cancer patients treated with VMAT, and found the corrCBCT (called CBCT_CC_ in their study) performed very similarly to the virtCT (called CT_V_ in their study), and both were adequate for dose recalculation on breast plans. Hamming et al. did note that the virtCT and its correction methods are not particularly useful for breast cancer patients, as the treated site is mostly uniform soft tissue with very few opportunities for low-density overrides. Taasti et al. [43] evaluated the feasibility of using corrCBCTs or virtCTs (Cor-sCT and DIR-sCT in their study) to monitor dose in lung cancer patients treated with IMPT. They concluded that both methods could reliably be used to trigger a vfCT (reCT in their paper) for further evaluation, as only 5% and 7% of patients had a false negative for the corrCBCT and virtCT, respectively. A false negative was defined as one or more clinical constraints failing on the reCT, but all constraints being satisfied on the sCT, resulting in only the reCT indicating the need for a plan adaptation [43]. O’Hara et al. [44] evaluated the accuracy of the corrCBCT (sCT3 in their study) for plan recalculation for head and neck cancer patients treated with photon VMAT. This study reported an increased dosimetric accuracy in the corrCBCT compared to older bulk density assignment methods due to the added HU and artifact corrections, but also noted that the corrCBCT struggled to accurately calculate dose at the boundary between high- and low-mass-density regions.

**Figure 9:**
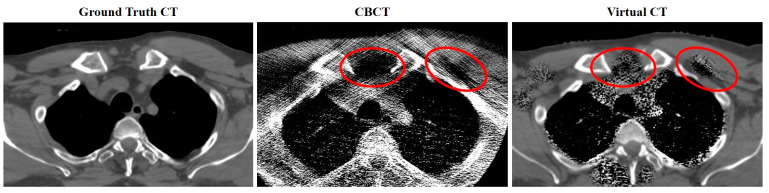
Demonstrating the effects of beam hardening and photon starvation artifacts between the shoulders. Areas circled in red demonstrate inaccurate tissue overrides in the virtual CT due to artifacts in the CBCT.

With regard to image accuracy, our study analyzed the accuracy and effectiveness of the algorithms by comparing the corrCBCT and virtCT to the process currently used clinically for evaluating dose throughout the treatment course, the vfCT. Our testing determined that both the corrCBCT and virtCT were significantly more accurate in terms of computing target coverage metrics than the vfCT. The greater dosimetric inaccuracy seen in the vfCT is due to setup differences between the vfCT scanning position and the actual treatment position, which is a limitation of the current workflow that was outlined in Figure 1. This is not experienced by the synthetic CTs, as they are based on the CBCT acquired prior to treatment. We also found that the virtCT was significantly more accurate than the corrCBCT (*p* = 0.0006) for calculating target coverage on high-risk CTVs, but did not demonstrate significance over the corrCBCT for standard-risk CTVs. The lesser performance of the virtCT for standard-risk CTVs relative to high-risk CTVs is likely due to photon starvation and beam hardening artifacts introduced lower in the neck and shoulder region, due to more bony structures and a more significant separation. The artifacts result in inappropriate low-density overrides near the lung and between the shoulders. The low-density overrides are mapped from the corrected CBCT to the virtual CT, so the inaccuracy of the corrCBCT in this region is matched in the virtCT. An example of this is shown in Figure 9.

Despite the improved dosimetric accuracy of the virtCT compared to the corrCBCT that was found in this analysis, there remains a clinical utility that is unique to the corrCBCT. The corrCBCT displays the actual anatomy that was imaged in the CBCT, while the virtCT relies on the accuracy of the DIR and low-density masking corrections. This means that even if the CT number mapping in the corrCBCT is less accurate than desired for dose recalculation, the image will still be useful for visual analysis of changes in patient anatomy and analyzing the accuracy of mapped contours. Due to the increased dose calculation accuracy of the virtCT and the superior anatomical accuracy of the corrCBCT, we recommend utilizing both image sets in a dose monitoring and adaptive planning workflow.

### 4.3. Workflow Improvement

The ability to reliably compute accurate proton plan doses on CBCT-based images enables an improved clinical process for evaluating patients for adaptive proton therapy. The proposed clinical workflow (shown in Figure 7) addresses multiple limitations in our current clinical workflow. Utilizing CBCT-based images for plan recalculation allows for dose evaluations between any fractions in the treatment course and does not require additional appointments or CT scans for the patient. The workflow also allows for more frequent assessment, making it more likely that plan adaptation will be performed appropriately and minimizing the number of fractions treated with a sub-optimal plan. Considering the unpredictable nature of anatomical changes for head and neck cancer, the ability to evaluate dose based on any fraction within the treatment course is critical. A study by Morgan et al. [1] summarizes reports of the changes in target volume throughout the treatment course for head and neck cancer and the heterogeneity of tumor response between patients within each study. Median tumor shrinkage rates ranged from 3 to 16% after week two, 7 to 48% after week four, and 6 to 66% after week seven. Studies also reported wide ranges of tumor response for patients within the same study, showing 73–79.6% reductions in tumor volume to 13–18.8% increases in tumor volume [1]. Given this inconsistency between patients, which has been demonstrated for patients at similar points in the treatment course being treated at the same institution and with identical planning practices and modalities, it is unsurprising that the review by Huiskes et al. [2] did not find any conclusions on optimal timing for adaptive radiation therapy in head and neck cancer patients. Therefore, it is essential to evaluate patients individually, with a frequency of evaluation that is not available with the current workflow.

Established thresholds for target coverage loss that trigger a review by the physician and physicist will also improve clinical efficiency, as evaluation doses in close agreement with the initial plan will not require review. Also, using the CBCT-based images allows setup inaccuracies to be noticed in dose evaluations, as the image will reflect the actual setup position from the treatments.

## 5. Conclusions

PBS proton therapy offers dosimetric advantages in treating head and neck cancer, and adaptive proton therapy is necessary for maintaining high plan quality over the treatment course. This work established target coverage thresholds for triggering further plan review in a high-frequency dose monitoring workflow for head and neck adaptive proton therapy, and tested two algorithms for producing CBCT-based synthetic CTs. The combination of established thresholds for plan review and the use of CBCT-based synthetic CTs within the treatment planning system improved clinical workflow for evaluating the delivered proton dose for any fraction. The improved workflow allows for more frequent and highly accurate interfraction dose evaluations that can detect anatomy and setup geometry changes that may lead to plan degradation, and decreases the need for routine mid-therapy vfCTs. These improvements will increase the likelihood of properly timing plan adaptations, maintaining higher plan quality over the treatment course, and improving overall patient experience.

## Figures and Tables

**Table 1:** Image characteristics of CBCT, planning CT, and verification CT.

	Image Dimensions (X, Y, Z)	Voxel Size (mm) (X, Y, Z)	Field-of-View (cm)	kVp	mAs
CBCT	512, 512, 110	0.54, 0.54, 2.50	26	100	110
Planning CT	512, 512, 387	1.17, 1.17, 1.00	60	140	Auto mAs
Verification CT	512, 512, 387	1.17, 1.17, 1.00	60	140	Auto mAs

X = right–left, Y = anterior–posterior, Z = inferior–superior (slice thickness).

**Table 2:** Summary of dosimetric accuracy for verification CT, corrected CBCT, and virtual CT.

	High-Risk CTV (N = 84)		Standard-Risk CTV (N = 84)	
	Average discrepancy from ground truth (%) |ΔD99|	*p*-value	Average discrepancy from ground truth (%) |ΔD99|	*p*-value
Verification CT	1.1 ± 1.4		1.8 ± 2.6	
Corrected CBCT	0.7 ± 0.7	0.04	0.5 ± 0.7	<0.0001
Virtual CT	0.4 ± 0.5	<0.0001	0.5 ± 0.6	<0.0001

Average values reported as mean ± standard deviation. *p*-value is calculated for corrCBCT and virtCT distributions relative to vfCT.

## Data Availability

Data supporting the findings of this study are available from the corresponding author, Yawei Zhang, upon reasonable request.
